# Prenatal Maternal Stress from a Natural Disaster Alters Urinary Metabolomic Profiles in Project Ice Storm Participants

**DOI:** 10.1038/s41598-018-31230-x

**Published:** 2018-08-28

**Authors:** Eric J. Paxman, Naveenjyote S. Boora, Douglas Kiss, David P. Laplante, Suzanne King, Tony Montina, Gerlinde A. S. Metz

**Affiliations:** 1 Canadian Centre for Behavioural Neuroscience, Department of Neuroscience, University of Lethbridge, 4401 University Dr W, Lethbridge, AB T1K 3M4 Canada; 2 Department of Chemistry and Biochemistry, University of Lethbridge, 4401 University Dr W, Lethbridge, AB T1K 3M4 Canada; 3 Douglas Hospital Research Centre, 6875 LaSalle Boulevard, Verdun, QC H4H 1R3 Canada; 4 Department of Psychiatry, McGill University, 845 Sherbrooke St. W., Montreal, QC H3A 0G4 Canada

## Abstract

Prenatal stress is known to epigenetically program offspring physiology and behaviour, and may become a risk factor for adult complex diseases. To gain insight into the underlying environment-gene interactions, we used proton nuclear magnetic resonance spectroscopy to analyze urinary metabolomes of male and female adolescents who were *in utero* during the 1998 Quebec Ice Storm. Metabolomic profiles in adolescent groups were found to be significantly different. Higher prenatal stress exposure generated alterations in metabolic pathways involved in energy metabolism and protein biosynthesis, such as branched-chain amino acid synthesis, alanine metabolism, and ketone body metabolism. Dysregulation of energy and protein metabolism suggests an increased risk of metabolic diseases like insulin resistance, diabetes, and obesity. These findings are consistent with prior observations of physiological phenotypes from this cohort. Understanding the impact of natural disasters on health risks will provide new and improved therapeutic strategies to mitigate stress-associated adverse health outcomes. Using metabolomic biomarkers may also assist in the prediction and prevention of these adverse outcomes.

## Introduction

Stress during pregnancy has a significant long-term impact on offspring physiology and behaviour^1–4^. Abnormally high levels of circulating maternal glucocorticoids and increased placental corticotropin-releasing hormone may cross the placenta to reach the fetal brain, leading to dysregulation of hypothalamic-pituitary-adrenal (HPA) axis activity and altered neuronal development^4,5^. Growing evidence suggests that these phenomena are associated with the pathophysiology of stress-related depression^6^. HPA axis programming due to fetal glucocorticoid overexposure has further potential lifetime consequences as a risk factor for complex adult diseases, including cardio-metabolic illnesses^7^, altered glucose and insulin metabolism, and adiposity^8,9^.

The association between HPA axis function and risk of metabolic diseases, such as diabetes and obesity, may be causally linked to altered genetic and epigenetic regulation. Epigenetics provides an additional layer of variation, mediating the relationship between internal and external environments and genotype^10^. Accordingly, prenatal maternal stress (PNMS) may alter DNA methylation^11,12^ and microRNA signatures in tissues of exposed offspring^13,14^. Genetic and epigenetic regulation affect cellular functions that ultimately will reflect an altered metabolic output. Hence, the metabolome, i.e., the sum of metabolites in an organism, arguably represents a direct measure of environment-gene interactions and associated phenotypes^15^.

Various biological fluids and tissues can be utilized to measure systemic metabolic changes. Urination is the primary method for excreting water-soluble metabolites from the body^16^. Urine sample accession is simple and non-invasive, making it ideal for metabolomic analyses. As the primary liquid by-product of cellular metabolism in humans and animals, urine is made up of numerous breakdown products of metabolic processes, such as nitrogenous wastes requiring clearance from the bloodstream. As such, urine can be expected to reflect metabolic activity from many organ systems. Since the brain accounts for up to 20% of metabolic activity in humans^17,18^, its waste products are significantly reflected in urine profiles.

Disease states and altered stress responses involve abnormal metabolic states that reflect altered cellular processes^10^. For example, adaptation to chronic stress facilitates visceral fat accumulation^19^ as adipocytes take up and catabolize glucocorticoids as a regulatory effect, justifying body fat gain as a response to stress^20^. In Project Ice Storm, 5½-year-old children of mothers who had experienced high disaster-related stress during pregnancy revealed increased risk of obesity^21^, which persisted into adolescence^22^. Studying natural disasters, such as exposure to the 1998 Quebec Ice Storm, allows for the isolation of the mother’s objective degree of exposure from genetic predispositions, socioeconomic biases, and from her own subjective level of distress^11^.

The goals of the present study were (a) to detect downstream metabolomic effects in male versus female adolescents resulting from prenatal maternal stress exposure using proton nuclear magnetic resonance (^1^H NMR) spectroscopy; (b) to differentiate between adolescents prenatally exposed to either low or high levels of PNMS based on presence and quantity of small-molecule metabolites in urine; and (c) to identify a subset of significantly altered metabolites and metabolic pathways that may be used to predict and determine future health status and disease risk in the study cohort. Metabolic signatures linked to perinatal programming of disease phenotypes may provide novel prognostic and diagnostic biomarkers and targets for therapeutic strategies.

## Results

Analyses using objective hardship as the predictor were based on 17 participants (9 males, 8 females) who were exposed *in utero* to high levels of objective hardship and 15 participants (9 males, 6 females) who were exposure *in utero* to low levels of objective hardship. Analyses using subjective distress as the predictor were based on samples obtained from 16 participants (9 males, 7 females) exposed *in utero* to high levels of subjective distress and 16 participants (9 males, 7 females) exposed *in utero* to low levels of subjective distress.

### Exploratory statistical analysis

Two hundred and fifty-five spectral bins were included in the analyses. Mann-Whitney U (MW) and Variable Importance Analysis Based on Random Variable Combination (VIAVC) tests were applied to each comparison group to identify which features (bins) led to observed group differences. These analyses resulted in the following subset of bins: objective hardship (male) (18 MW, 7 VIAVC, and 4 common bins); subjective distress (male) (17 MW, 10 VIAVC, and 2 common bins); objective hardship (female) (8 MW, 4 VIAVC, and 1 common bin); and subjective distress (female) (33 MW, 2 VIAVC, and 2 common bins). Multivariate statistical tests were initially performed using all bins, with no separation of groups observed (data not shown). Group separation was observed when only the bins identified as significant by either VIAVC or MW tests were used in PCA and PLS-DA analyses.

**Figure 1:**
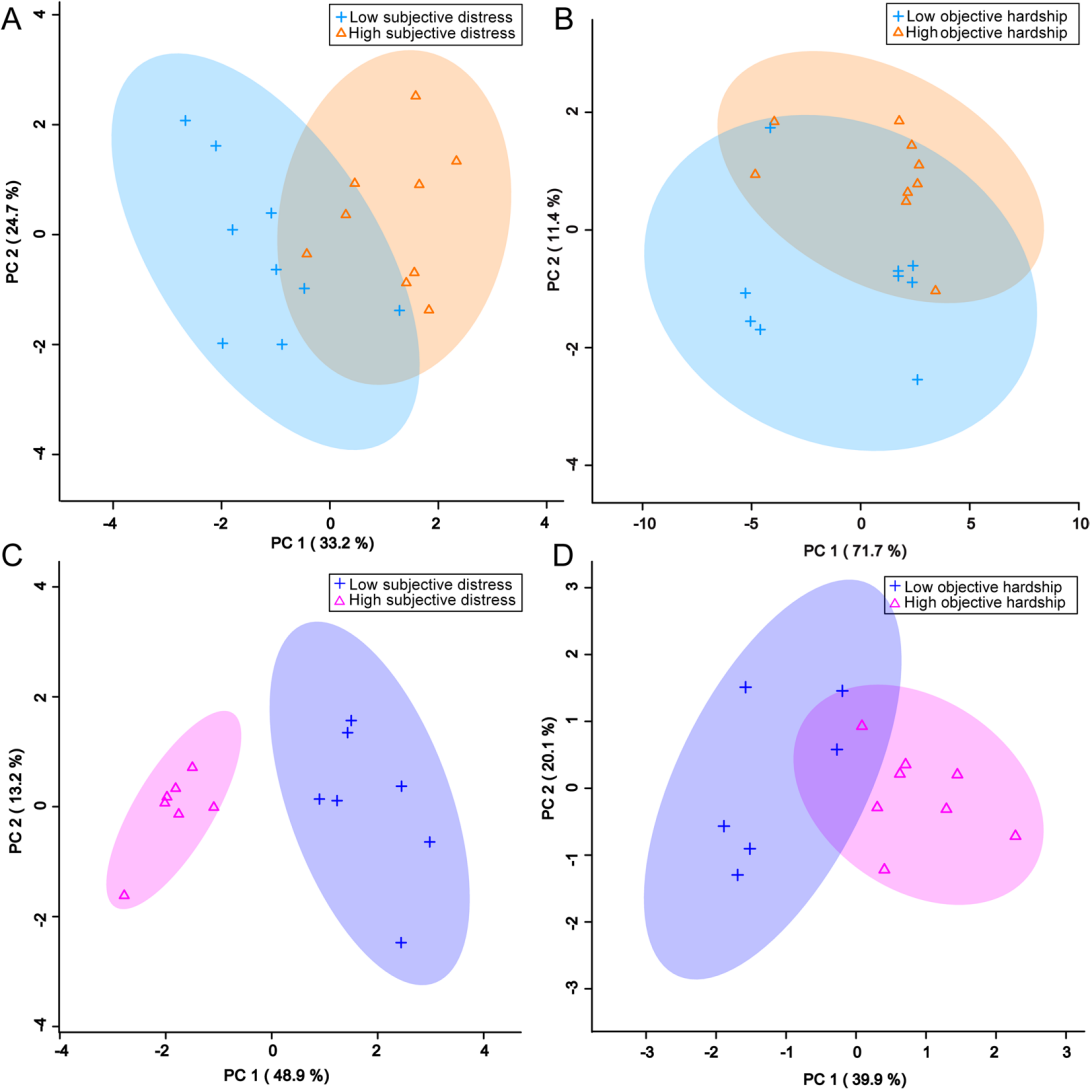
PCA plots showing statistically significant unsupervised separation for male (**A**,**B**) and female (**C**,**D**) adolescents exposed in utero to high or low levels of PNMS. (**A**,**C**) Subjective distress measured by the Impact of Events Scale – Revised (IES-R) in mothers exposed to stress during pregnancy. (**B**,**D**) Objective hardship measured by the Storm 32 survey (ST32) in mothers exposed to stress during pregnancy. Each triangle or cross represents one individual under study, plotted using a list of urinary metabolites found to be statistically significant by either MW or VIAVC testing. X and Y axes show principal components with brackets indicating percent variance.

**Figure 2:**
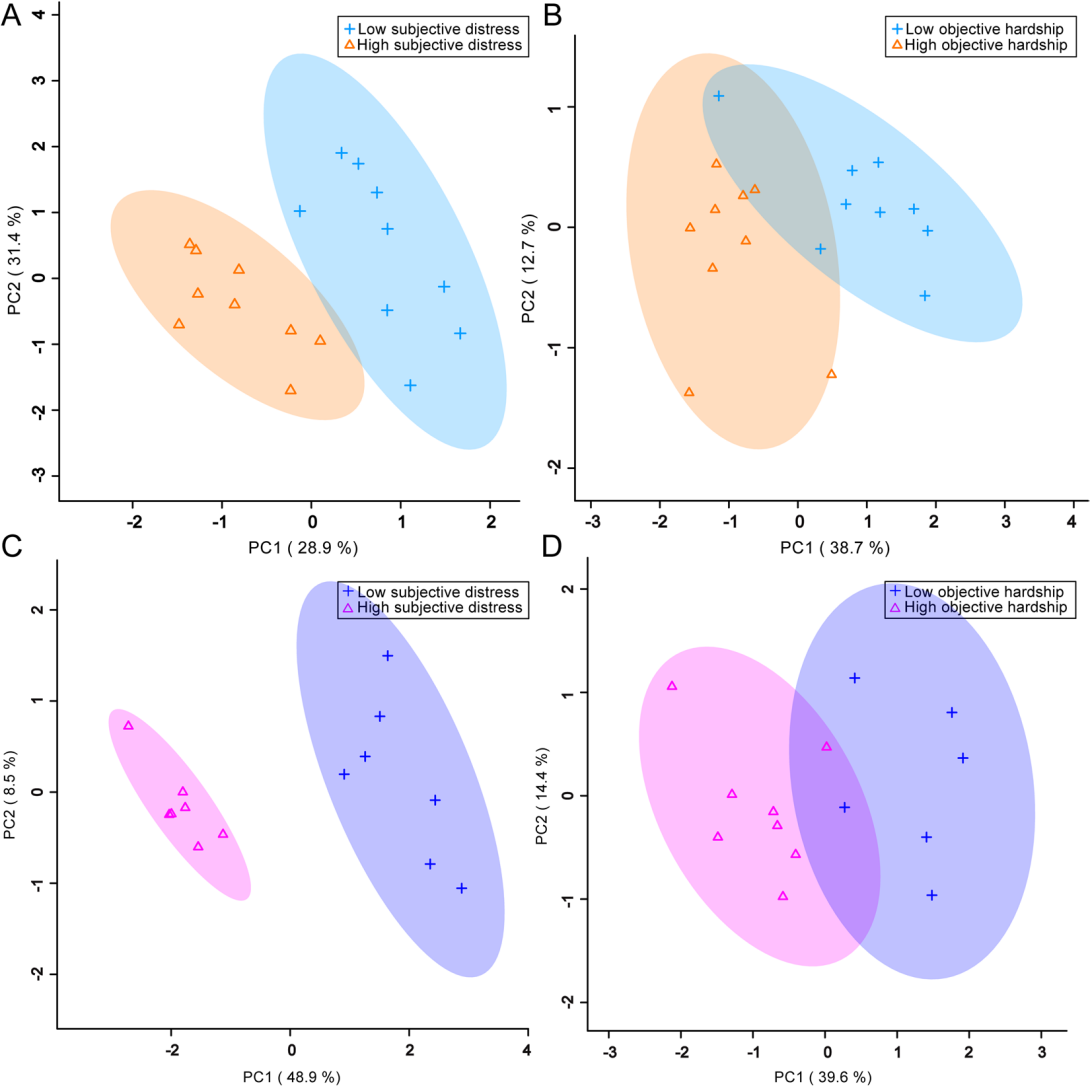
PLS-DA plots showing statistically significant supervised separation for male (**A**,**B**) and female (**C**,**D**) adolescents exposed to high or low levels of PNMS. (**A**,**C**) Subjective distress during pregnancy. (**B**,**D**) Objective hardship during pregnancy. Each triangle or cross represents one individual under study, plotted using a list of urinary metabolites found to be statistically significant by either MW or VIAVC testing. X and Y axes show principal components with brackets indicating the percent variance.

In males, the unsupervised PCA separation was significant, particularly when split by the level of subjective distress exposure (Fig. 1A,B). Subsequent supervised PLS-DA separations were significant and supported the PCA findings (Fig. 2A,B). For females, significant unsupervised separation was observed when the sample was split by *in utero* levels of both maternal subjective distress and objective hardship (Fig. 1C,D). Significant supervised separation for females was also observed (Fig. 2C,D). Double cross-validation and permutation tests validated the observed supervised separation results as a function of subjective distress (male p = 0.034, female p < 0.001) and objective hardship exposure levels (male p = 0.01, female p = 0.011).

**Figure 3:**
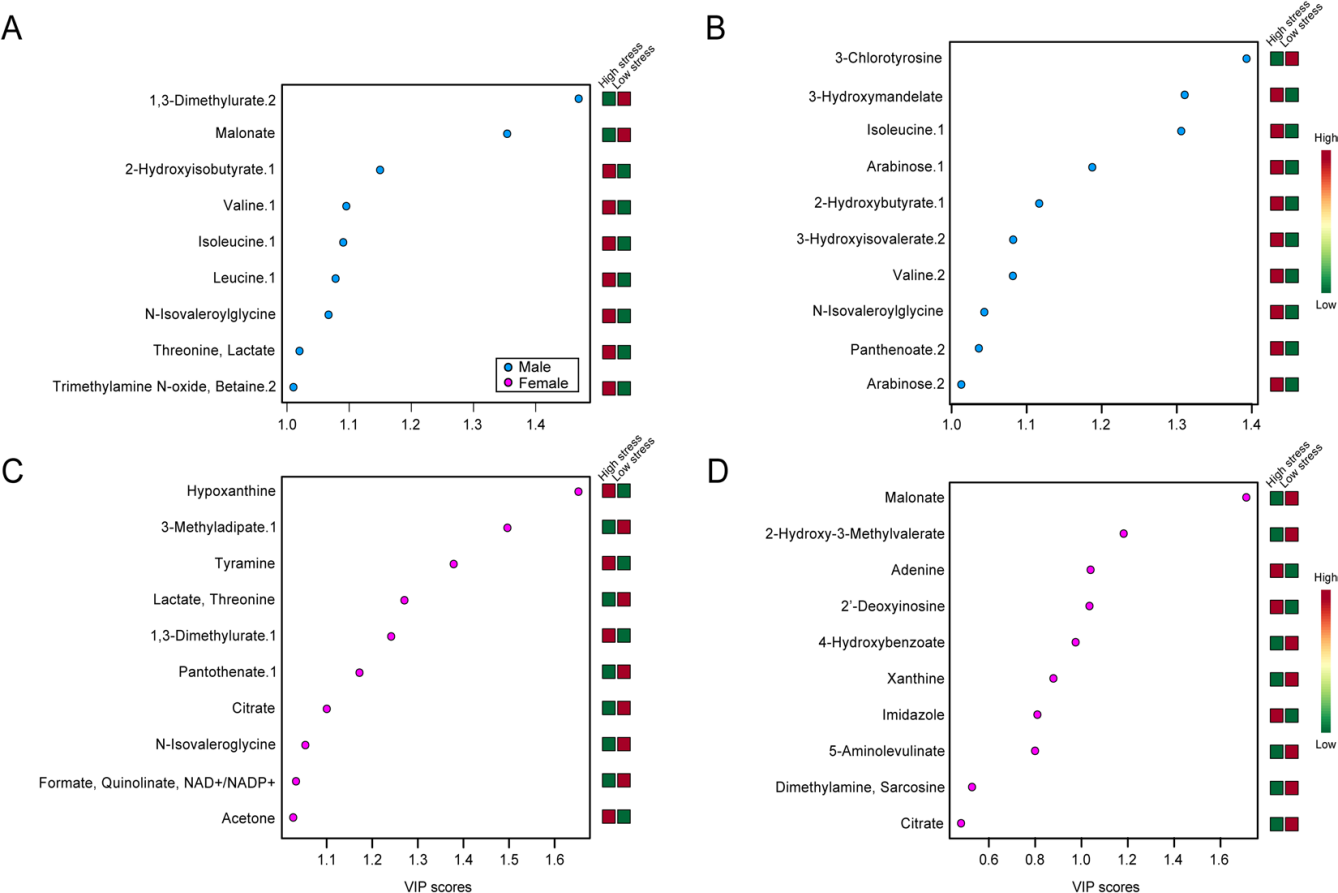
VIP plots of subjective distress and objective hardship, showing the relative contribution of metabolites. (**A**) Male subjective distress. (**B**) Male objective hardship. (**C**) Female subjective distress. (**D**) Female objective hardship. High VIP values indicate greater contribution of these metabolites to group separation, shown in PLS-DA plots. Green and red boxes indicate relative metabolite concentration. A VIP score of 1.0 is considered able to discriminate between two phenotypes.

Variable Importance in Projection (VIP) plots (Fig. 3) indicate which variables contribute most to significant supervised separation. In males, 1,3-dimethylurate, malonate, and 2-hydroxybutyrate contributed most to the separation when subjective distress was included (Fig. 3A), with VIP scores of 1.47, 1.35, and 1.15 (Table 1). When objective hardship was included, 3-chlorotyrosine, 3-hydroxymandelate, and isoleucine contributed most (Fig. 3B), with VIP scores of 1.39, 1.31, and 1.31, respectively (Table 1). In females, the top three compounds that contributed to separation when subjective distress was included were hypoxanthine, 3-methyladipate, and tyramine (Fig. 3C), with VIP scores of 1.65, 1.50, and 1.38 (Table 2). The top three compounds contributing to group separation when objective hardship was included (Fig. 3D) were malonate, 2-hydroxy-3-methylvalerate, and adenine, with VIP scores of 1.71, 1.18, and 1.04, respectively (Table 2). In males, 22/27 (81%) unique, significant metabolites were up-regulated in high compared to low PNMS, with the remaining metabolites being down-regulated (Table 1). In contrast, in females 27/35 (77%) unique, significant metabolites were down-regulated in high compared to low PNMS, with the remainder being up-regulated (Table 2).

**Table 1:** P-values of urinary metabolites found to be significant in male subjective distress and objective hardship groups (IESR and ST32, respectively) in either a Mann-Whitney U test, the Variable Importance Analysis based on random Variable Combination (VIAVC), or both.

Group	Metabolite	NMR Chemical Shift Range of bin (ppm)	Mann-Whitney U Test	VIAVC	VIP Score	Regulation
Male Subjective Distress (IESR)	1,3-Dimethylurate.2^‡^	3.317–3.2958	—	2.94E-88	1.47	Down
	Malonate^‡^	3.120–3.104	—	2.17E-53	1.35	Down
	2-Hydroxyisobutyrate.1^‡^	1.368–1.354	3.99E-03	1.68E-09	1.15	Up
	Valine.1	1.005–0.998	1.85E-02	5.19E-01	1.1	Up
	Isoleucine.1^**†**^	0.998–0.993	1.88E-02	2.37E-04	1.09	Up
	Leucine.1^‡^	0.965–0.958	2.76E-03	2.27E-07	1.08	Up
	N-Isovaleroylglycine^‡^	0.958–0.948	3.99E-03	5.23E-06	1.07	Up
	Threonine, Lactate^‡^	1.354–1.338	3.99E-03	1.59E-10	1.02	Up
	Trimethylamine N-Oxide, Betaine.2^‡^	3.254–3.240	4.00E-02	4.99E-17	1.01	Up
	2,6-Dihydroxybenzoate	6.495–6.439	1.88E-02	1.78E-03	0.96	Up
	Leucine.2^†^	0.971–0.965	1.42E-02	2.96E-03	0.96	Up
	5-Hydroxyindole-3-Acetate	7.047–7.024	—	3.54E-01	0.94	Up
	Leucine.3	0.979–0.971	2.43E-02	1.93E-04	0.94	Up
	Valine.2^†^	1.054–1.044	3.15E-02	2.01E-02	0.92	Up
	1,3-Dimethylurate.1^‡^	3.349–3.317	4.00E-02	4.24E-13	0.89	Down
	Alanine	1.476–1.460	—	1.35E-01	0.88	Up
	3-Hydroxy-3-Methylglutarate	1.338–1.313	3.15E-02	2.53E-12	0.87	Up
	Pantothenate.2^‡^	0.912–0.903	1.06E-02	1.26E-03	0.84	Up
	Valine.3^‡^	1.013–1.005	1.42E-02	1.04E-03	0.8	Up
	3-Methyladipate.6	0.919–0.912	2.44E-02	1.35E-02	0.76	Up
	2-Hydroxybutyrate.2^‡^	1.388–1.368	4.00E-02	1.89E-02	0.75	Up
	3-Hydroxyisovalerate.2^†^	1.285–1.265	—	1.49E-03	0.66	Up
	Betaine.1	3.905–3.881	—	8.35E-15	0.64	Up
Male Objective Hardship (ST32)	3-Chlorotyrosine	6.964–6.953	3.99E-03	1.40E-05	1.39	Down
	3-Hydroxymandelate	6.925–6.913	3.99E-03	1.27E-04	1.31	Up
	Isoleucine.1^†^	0.998–0.993	5.64E-03	2.04E-03	1.31	Up
	Arabinose.1	4.528–4.511	1.42E-02	1.52E-03	1.19	Up
	2-Hydroxyisobutyrate.1^‡^	1.368–1.354	1.42E-02	7.71E-06	1.12	Up
	3-Hydroxyisovalerate.2^†^	1.285–1.265	3.15E-02	3.63E-08	1.08	Up
	Valine.2^†^	1.054–1.044	3.15E-02	1.50E-06	1.08	Up
	N-Isovalerylglycine^‡^	0.958–0.948	1.88E-02	1.50E-07	1.04	Up
	Pantothenate.2^‡^	0.912–0.903	5.64E-03	6.54E-04	1.04	Up
	Arabinose.2	4.540–4.528	1.42E-02	4.31E-05	1.01	Up
	Leucine.1^‡^	0.965–0.958	3.15E-02	3.01E-04	0.96	Up
	Isoleucine.2^‡^	1.038–1.013	2.44E-02	4.87E-07	0.9	Up
	Leucine.2^†^	0.971–0.965	3.99E-03	7.65E-02	0.88	Up
	Pyruvate, Succinate, Oxalacetic Acid	2.402–2.366	3.15E-02	3.30E-07	0.84	Up
	Valine.3^‡^	1.013–1.005	2.44E-02	3.27E-02	0.79	Up
	N6-Acetyllysine	1.973–1.955	3.99E-03	1.29E-03	0.65	Up
	Tryptophan	7.746–7.711	—	3.10E-03	0.36	Up
	Methylsuccinate	1.067–1.054	3.99E-03	1.00E + 00	0.25	Down

VIP scores, shown in descending order, correspond to Fig. 3A,B. Metabolite regulation is shown as a function of relative concentration in high-PNMS individuals. Metabolites for which more than one NMR resonance peak was identified as significant are represented as metabolite.1, metabolite.2, … metabolite.n. ^**†**^Indicates metabolites that were present in both male comparisons while ^‡^indicates metabolites that were present in both sexes and multiple comparisons.

**Table 2:** P-values of urinary metabolites found to be significant in female subjective distress and objective hardship groups (IESR and ST32, respectively) in either a Mann-Whitney U test, the Variable Importance Analysis based on random Variable Combination (VIAVC), or both. VIP scores, shown in descending order, correspond to Fig. 3C,D.

Group	Metabolite	NMR Chemical Shift range of bin (ppm)	Mann-Whitney U Test	VIAVC	VIP Score	Regulation
Female Subjective Distress (IESR)	Hypoxanthine	8.200–8.165	2.62E-02	3.73E-83	1.65	Up
	3-Methyladipate.1	0.876–0.823	5.83E-04	1.65E-80	1.5	Down
	Tyramine	7.206–7.154	1.11E-02	1.44E-17	1.38	Up
	Threonine, Lactate^‡^	1.354–1.338	4.08E-03	5.10E-59	1.27	Down
	1,3-Dimethylurate.1^‡^	3.349–3.317	2.62E-02	1.87E-42	1.24	Up
	Pantothenate.1	0.902–0.876	2.33E-03	3.30E-62	1.17	Down
	Citrate^†^	2.545–2.520	3.79E-02	5.04E-02	1.1	Down
	N-Isovalerylglycine^‡^	0.958–0.948	1.11E-02	1.98E-13	1.05	Down
	Formate, Quinolinate, NAD+/NADP+	8.457–8.370	1.75E-02	7.69E-05	1.03	Down
	Acetone	2.225–2.209	1.11E-02	2.50E-14	1.03	Up
	Leucine.1^‡^	0.965–0.958	2.62E-02	5.45E-08	0.99	Down
	Valine.3^‡^	1.013–1.005	6.99E-03	1.80E-07	0.95	Down
	2-Hydroxyisobutyrate.2^‡^	1.388–1.368	6.99E-03	4.04E-12	0.91	Down
	Pantothenate.2^‡^	0.912–0.903	6.99E-03	1.20E-02	0.9	Down
	Acetoacetate	2.256–2.225	1.11E-02	1.66E-18	0.89	Down
	UDP-Galactose, UDP-Glucose, UDP-Glucuronate	4.396–4.365	4.08E-03	2.26E-18	0.85	Down
	3-Hydroxyisovalerate.1	2.366–2.359	2.62E-02	1.40E-06	0.82	Down
	3-Methyladipate.2	2.319–2.310	1.75E-02	1.50E-01	0.79	Down
	3-Methyladipate.3	0.928–0.919	6.99E-03	2.35E-03	0.79	Down
	2-Hydroxyisobutyrate.1^‡^	1.368–1.354	1.11E-02	1.26E-05	0.77	Down
	Isoleucine.2^‡^	1.038–1.013	1.75E-02	1.19E-01	0.76	Down
	Trimethylamine N-Oxide, Betaine.2^‡^	3.254–3.240	3.79E-02	2.17E-05	0.74	Up
	5-Aminolevulinate^†^	2.814–2.793	2.62E-02	1.78E + 00	0.74	Down
	Unknown Singlet	1.671–1.648	1.11E-02	2.91E-02	0.74	Down
	3-Methyladipate.4	1.909–1.835	3.79E-02	9.09E-08	0.71	Down
	Arginine	1.817–1.671	6.99E-03	5.80E-29	0.69	Down
	3-Methyladipate.5	2.017–2.010	2.62E-02	3.57E-03	0.62	Down
Female Objective Hardship (ST32)	Malonate^‡^	3.120–3.104	1.27E-02	5.05E-138	1.71	Down
	2-Hydroxy-3-Methylvalerate	1.188–1.178	2.93E-02	1.31E-07	1.18	Down
	Adenine	8.072–8.044	2.94E-02	3.66E-04	1.04	Up
	2′-Deoxyinosine	8.319–8.292	—	1.07E-05	1.03	Up
	4-Hydroxybenzoate	7.772–7.746	2.00E-02	1.51E-02	0.97	Down
	Xanthine	7.817–7.772	4.26E-02	1.29E + 00	0.88	Down
	Imidazole	7.009–6.995	2.93E-02	1.99E-02	0.81	Up
	5-Aminolevulinate^†^	2.814–2.793	4.26E-02	1.52E+00	0.8	Down
	Dimethylamine, Sarcosine	2.714–2.637	—	7.86E-08	0.53	Down
	Citrate^†^	2.545–2.520	—	1.73E-02	0.48	Down

Metabolite regulation is shown as a function of relative concentration in high-PNMS individuals. Metabolites for which more than one NMR resonance peak was identified as significant are represented as metabolite.1, metabolite.2, … metabolite.n. ^†^Indicates metabolites that were present in both female comparisons while ^‡^indicates metabolites that were present in both sexes.

### Functional analysis

**Figure 4:**
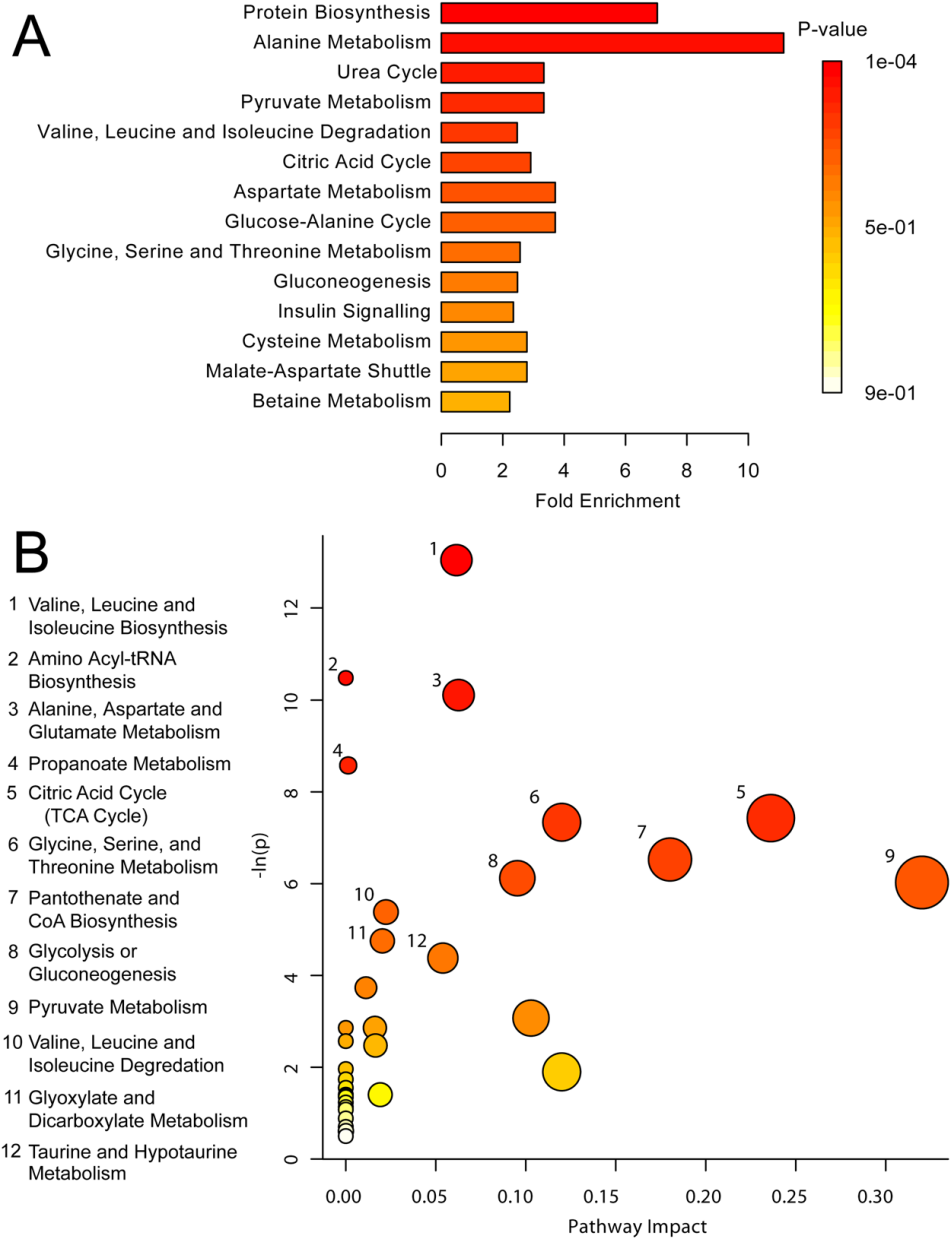
(**A**) MSEA plot in male offspring of stressed mothers. (**B**) Metabolomic Pathway Analysis showing all matched pathways according to p-values from pathway enrichment analysis and pathway impact values in males. A higher value on the y-axis indicates a lower p-value. The x-axis gives the Pathway Impact. Only metabolic pathways with p < 0.015 are labeled. This figure was created using the lists of metabolites identified as significant for either MW or VIAVC testing, for both male comparison groups.

**Figure 5:**
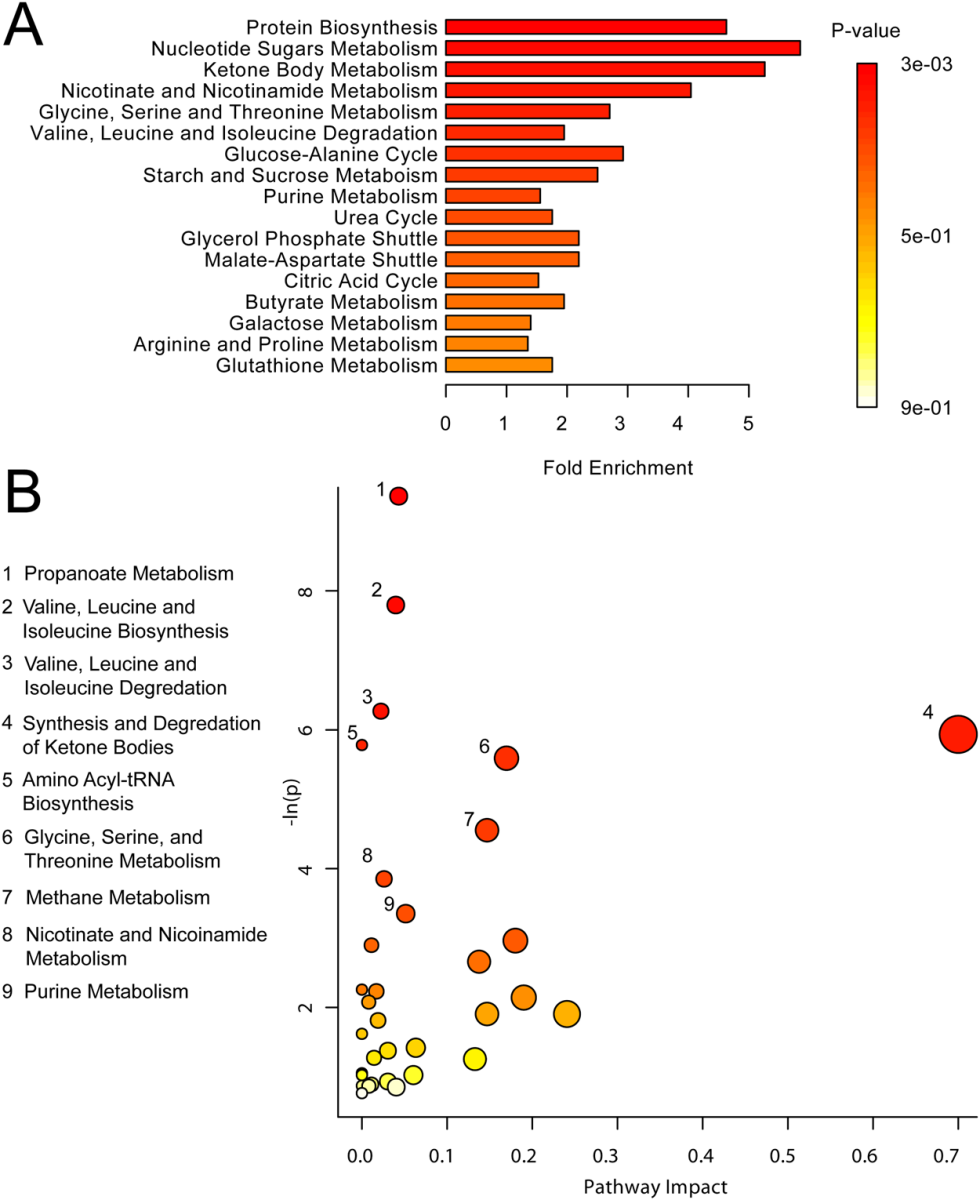
(**A**) MSEA plot in female offspring of stressed mothers. (**B**) Metabolomic Pathway. Higher value on the y-axis gives a lower p-value. The x-axis gives the Pathway Impact, which correlates to the number of metabolite hits in a particular pathway. Only metabolic pathways with p < 0.05 are labeled. This figure was created using the lists of metabolites identified as significant for either MW or VIAVC testing, for both female comparison groups.

Metabolic sets were predicted to be changed in the case of dysfunctional enzymes, using a genome-scale network model of human metabolism (Figs 4 and 5). High PNMS exposure in male adolescents significantly affected branched chain amino acid (BCAA; leucine, isoleucine, and valine) biosynthesis (p < 0.01) (Fig. 4A). Additionally, numerous energy metabolism systems were altered, including pathways in the citric acid cycle (p < 0.001) and gluconeogenesis (p < 0.01) (Fig. 4B). High PNMS exposure in females also had the greatest impact on protein biosynthesis (p < 0.01), as well as nucleotide sugar metabolism (p < 0.01) and ketone body metabolism (p = 0.016) (Fig. 5A). This is supported by the pathway topology analysis (Fig. 5B), showing significant effects on valine, leucine, and isoleucine (BCAA) biosynthesis/degradation (p < 0.01), propanoate metabolism (p < 0.001), and ketone body biosynthesis/degradation (p > 0.01).

## Discussion

Stress is one of the most critical determinants of lifetime health. Here we show (i) that adolescents whose mothers experienced high or low objective or subjective distress during pregnancy can be clearly identified by profiling small-molecule metabolites in urine; and (ii) that urinary metabolites are significantly altered in adolescents born to mothers who experienced high objective and/or subjective stress during a natural disaster, compared to those who experienced low objective and/or subjective stress. The majority of the metabolites found to be significantly altered belong to metabolic pathways involved in energy metabolism and protein biosynthesis, supporting a link between early adverse life events and risk of metabolic illness later in life^23^.

Forty-nine metabolites known to be present in urine^15^ were identified as significantly related to the level and type of PNMS experienced *in utero*. The class separation may reflect underlying epigenetic changes and associated cellular functions that manifest as downstream alterations to the metabolome. Gender-related fetal differences in genetics, physiology or metabolism may also lead to differential downstream effects manifesting in the metabolome. While our results show opposite effects in protein metabolism among males and females, particularly in regulation of branched-chain amino acids (BCAAs) and protein biosynthesis, the present results also suggest gender differences in lipid and ketone body metabolism, nucleotide sugar metabolism, and energy metabolism. These findings are further supported by exercise physiology studies showing that females oxidize proportionately more fat and less carbohydrate than males^24,25^.

Almost all metabolites that obtained a significant VIP score for both males and females are involved in energy metabolism. Abnormal levels of metabolites involved in the Krebs cycle, such as citrate and malonate, can lead to energy deficiency, which is an important factor in fatigue, one of the most frequently represented symptoms in major depressive disorder^26^. Interestingly, BCAAs, which are critical to human life and particularly involved in stress responses, energy, immunity, and muscle metabolism^27^, are highly represented in the VIP plots for males, and in pathway analyses for both males and females.

In both genders, malonate, which is a competitive inhibitor of succinate dehydrogenase in the electron transport chain (ETC)^28^, was highly significant in contributing to unsupervised and supervised separations. In females, the purine derivative and adenosine reaction intermediate hypoxanthine, as well as the purine base adenine, were found to significantly contribute to group separation. Both metabolites are involved in purine and energy metabolism, supporting gender differences in these systems^29^. Hypoxanthine has been found to increase significantly in blood serum of obese individuals during exercise^30^. In contrast, 3-hydroxymandelate and 3-chlorotyrosine were identified as highly important metabolites in the unsupervised and supervised separation seen in males. These appear to be breakdown products or intermediates in major oxidation pathways^31,32^. Interestingly, 3-chlorotyrosine has been shown to be markedly elevated in low-density lipoprotein isolated from atherosclerotic intima^33^, suggesting a link to atherogenesis in prenatally stressed male offspring.

MSEA was used to identify patterns of metabolite concentration changes in a biologically meaningful framework^34^. The most significant and consistent pathway was protein biosynthesis, with 6 metabolite hits in males and 5 metabolite hits in females. Thus, the formation and breakdown of both proteins and amino acids are involved in the response to high PNMS exposure. Significant pathways in the female MSEA, apart from protein biosynthesis, included ketone body metabolism, nucleotide sugar metabolism, and nicotinate and nicotinamide metabolism. Nicotinate and nicotinamide play essential roles in respiration, glycolysis, and fatty acid synthesis, acting as coenzymes and ADP-ribose donors^35,36^. Disruption in protein biosynthesis and energy metabolism supports the finding that adolescents who were exposed *in utero* to high degrees of objective hardship during the 1998 Quebec Ice Storm are at higher risk for developing obesity, independent of size at birth and maternal characteristics such as height or body mass index^21,22^. Furthermore, diabetes is the most common pathological process known to cause ketone body dysregulation in the blood^9,37^ and the present data support a causal role for PNMS.

Consistent with MSEA results and VIP scores, both the male and female pathway topology analyses reveal changes in valine, leucine, and isoleucine synthesis and degradation. In combination with up-regulation of BCAAs in males, this draws attention to a possible metabolic dysregulation of BCAAs in male adolescents of high PNMS. Circulating levels of BCAAs have been shown to be increased in obese individuals and are associated with poor metabolic health and future insulin resistance, suggesting the possibility that these amino acids contribute to pathogenesis of obesity and diabetes^38,39^. Previous research has also shown that pathways related to energy and lipid metabolism are consistently associated with Bipolar Disorder, Major Depressive Disorder, and Schizophrenia^40^.

The alanine pathway, which involves the breakdown of pyruvate and some dipeptides, was significantly altered between high and low stress male adolescents. Alanine is one of the most important amino acids released by muscle tissue, acting as a major energy source and an important regulator in glucose metabolism, lymphocyte production, and immunity^41,42^. Alterations in the alanine cycle that increase levels of serum alanine aminotransferase (ALT) have been linked to the development of type II diabetes^43^. Altogether, the results of this study suggest that adolescents whose mothers experienced high stress during pregnancy, particularly males, may be at an increased risk for developing obesity and diabetes later in life.

The present findings indicate that metabolomic signatures assessed by ^1^H NMR spectroscopy serve as clinically accessible predictive and diagnostic biomarkers of disease. Compared to other methods, NMR spectroscopy appears to be the method of choice for global, untargeted metabolomic analysis of urine^15^, as it permits measurement of the largest number of metabolites (209, compared to 179 in gas chromatography - mass spectrometry and 127 in liquid chromatography - mass spectrometry), and yields the largest chemical diversity. Furthermore, NMR is non-destructive, so samples can be saved and re-used for further analysis. Lastly, urine is an ideal biofluid for stress metabolomic studies since it is very easy to obtain, non-invasive, does not easily transmit infectious diseases, and it contains many clearly identifiable metabolites (209 compared to 53 in cerebrospinal fluid and 49 in blood).

On a cautionary note, the biomarker potential of urinary metabolomics analyses may be limited by varying metabolite concentration (scaled to creatinine) of the average compound in normal human urine by ± 50%, with some varying by as much as 350%^15^. These ranges are determined by gender, age, genetic background, diet, and activity level of the subject^44–46^. In this study, however, adolescents were separated by gender, were approximately the same age, and were from the same geographical region, mitigating much of the normal variance in metabolite quantities seen across individuals.

Using an isolated traumatic stressor, the 1998 Quebec Ice Storm, experience-dependent biomarker signatures resulting from PNMS were detected as downstream metabolomic changes using ^1^H NMR spectroscopy. Here, we accurately differentiate pathologically high and low prenatal stress groups based on a subset of significantly altered metabolites and/or metabolic pathways, which are potentially linked to metabolic illness, such as insulin resistance, diabetes, and obesity. The results support long-lasting metabolomic differences in males and females, such as ketone body production and energy metabolism. Some similarities between high-stress male and female adolescents were also identified, which include alterations in BCAA biosynthetic pathways. Possibilities exist to correlate future work, such as body composition analyses and other health outcomes, with the findings of this study.

## Methods and Materials

### Study design

#### Sample development

During the first week of January 1998, the Southern region of the Canadian province of Quebec experienced a severe ice storm that decimated the power grid, leaving more than 1.5 million households without electricity for up to 45 days. Damage from the storm made it one of the costliest disaster in Canadian history and was responsible for 27 deaths.

To identify and recruit women who met criteria for inclusion into the study (pregnant on January 9, 1998 or became pregnant during the following 3 months after the storm, at least 18 years of age, and spoke fluent French), physicians who delivered babies from four regional hospitals were asked to mail out an initial recruitment questionnaire to their eligible patients on June 1, 1998. Mothers and their children have subsequently been assessed several times: at the ages of 6 months, 2, 4, 5½, 8½, 9½, 11½, 13½, 16½, and 18½ years. The participants in the present study included 18 males and 14 females at the age of 16–17 years.

This study was approved by the Research Ethics Boards of the Douglas Hospital Research Center and the University of Lethbridge in accordance to the Canadian Best Practices for Health Research Involving Children and Adolescents. We obtained written informed consent from parents and written informed assent from adolescents.

#### Assessment of objective hardship and subjective distress

Objective hardship was estimated using the mothers’ responses to questions about their ice storm experiences from categories of exposure: Threat, Loss, Scope, and Change^47^. A total objective stress score (STORM32) was calculated by summing scores from all four dimensions using McFarlane’s approach^48^. The mothers’ subjective distress related to the ice storm was assessed using a validated French adaptation^49^ of the widely used Impact of Event Scale – Revised (IES-R)^50^. The 22-item instrument provides scores for symptoms in three scales relevant to post-traumatic stress disorder: Intrusive Thoughts, Hyperarousal, and Avoidance^50^. The total score was used. High and Low Objective and Subjective PNMS groups were obtained by splitting the distributions at the median.

### Metabolomics assessment

#### Sample collection and preparation

Thirty-two male (n = 18) and female (n = 14) participants were instructed to obtain a urine sample midstream at their first passage of the day, which was subsequently stored at −80 °C until further processing. One female sample was removed after discovering elevated levels of glucose in the urine using Chemstrip 9 (Roche Diagnostics, Indianapolis, IN, USA). In total, 450 µL of urine and 150 µL of phosphate buffer were transferred into 1.5 mL centrifuge tubes. The sample/buffer solution was vortexed and then centrifuged at 12,000 g for 5 minutes at 4 °C to precipitate and pellet any particulate matter. Following centrifugation, 550 µL of supernatant was transferred to a 5-mm NMR tube to be analyzed immediately. Phosphate buffer was prepared as a 4:1 ratio of KH_2_PO_4_:K_2_HPO_4_ in a 4:1 H_2_O:D_2_O solution to obtain a final concentration of 0.5 M^51^. The D_2_O contained 0.05% v/v trimethylsilylpropanoic acid (TSP) as a chemical shift reference for ^1^H NMR spectroscopy. Sodium azide (NaN_3_, 0.03% w/v) was added as an antimicrobial agent, and total buffer pH was titrated to 7.4 using 3 M HCl.

#### NMR data acquisition and processing

Spectra were collected on a 700 MHz Bruker Avance III HD spectrometer (Bruker, ON, Canada) as described previously^52^. All measurements were recorded using a Bruker triple resonance TBO-Z probe with the outer coil tuned to the nuclei of ^1^H, ^31^P and ^2^H and the inner coil tuned to the ^13^C nucleus. The 1-D NOESY gradient water suppression pulse sequence noesygpr1d with 10 ms mixing time was used (Bruker). Samples were run for 128 scans, with a total acquisition size (TD) of 128k, a spectral window (SW) of 20.5 ppm, a transmitter offset (o1p) of 4.7 ppm, and a recycle delay of 4 seconds. The Bruker automation (au) program “pulsecal” was utilized on each sample prior to data acquisition to guarantee that the 90-degree pulse was calibrated correctly, which ensures quantitative and comparable data across samples. Spectra were zero filled to 256k, automatically phased and baseline corrected, and line-broadened by 0.3 Hz. Processed spectra were exported to MATLAB (MathWorks, MA, USA) and binned using Dynamic Adaptive Binning^53^. The dataset was then normalized using the Constant Sum method, where each spectrum is set to have a unit total area and each data point (bin) is a fraction of the total spectral integral (with the regions corresponding to water and urea removed). The data set was then Pareto-scaled (mean-centered and divided by the square root of each variable’s standard deviation) to reduce the influence of intense peaks, while emphasizing weaker ones. All peaks were referenced to TSP (0.00δ).

### Statistical analyses

Two hundred and fifty-five spectral bins were first analyzed for all comparison groups and deemed significant or non-significant using a decision tree algorithm^54^ and Mann-Whitney U test. All p-values from this algorithm were Bonferroni-Holm corrected for multiple comparisons. Data visualization was conducted using Principal Component Analysis (PCA) and Partial Least Squares Discriminant Analysis (PLS-DA) using Metaboanalyst^34,55–63^. Double cross-validation and permutation testing (minimum 1,000 iterations) were performed to verify and support all statistically significant PLS-DA results^64,65^. Variable Importance in the Projection (VIP) plots were made using the weighted sum of squares of the PLS loadings, based on explained Y-variance in each dimension.

Variable Importance Analysis based on random Variable Combination (VIAVC) was used^66^ as an additional feature/bin selection method. VIAVC systematically resamples variables to reveal any synergistic effects that may exist between seemingly unimportant variables^66^, which is unaccounted for in univariate statistical tests. By combining random permutations of variable inclusions with ten-fold double cross validation, and by using the Area Under the Curve (AUC) of a Receiver-Operator Characteristic (ROC), VIAVC determines an optimal subset of variables that have led to the most substantial group differences. The optimal subset of variables is determined using the complete cross validation methods outlined by Westerhuis *et al*.^64^. This method sets aside one set of samples as the independent test (validation) set and the other samples are included in the calibration set. The calibration set is used to form the multivariate model which is tested for predication accuracy against the samples held back in the validation set. This procedure is repeated multiple times until all samples have been chosen to be a part of the validation set at least once. The reported *p-*values for the optimal subset are calculated from a t-test of distribution scores based on whether a particular metabolite was included in the model.

Biological significance of important metabolites was investigated using the Metabolite Set Enrichment Analysis (MSEA) and Pathway Topology Analysis through Metaboanalyst. Metabolic pathway analyses identified the most relevant pathways^62^ based on the Human Pathway Library and the Over Representation Analysis algorithm selected, using a hypergeometric test.

### Metabolite identification

The Human Metabolome Database^42,67,68^ and the Chenomx 8.2 NMR Suite (Chenomx Inc., Edmonton, Alberta, Canada) were used for spectral deconvolution of biofluid samples into individual components.
